# Cytogenetic Abnormalities in Lymphocytes from Victims Exposed to Cobalt-60 Radiation

**DOI:** 10.3390/ijms140917525

**Published:** 2013-08-27

**Authors:** Jia Cao, Jing Zhang, Yan Wang, Li Qing Du, Chang Xu, Qin Wang, Jian Xiang Liu, Xu Su, Fei Yue Fan, Qiang Liu, Sai Jun Fan

**Affiliations:** 1 Institute of Radiation Medicine, Chinese Academy of Medical Sciences and Peking Union Medical College, Tianjin 300192, China; E-Mails: jillpumc@gmail.com (J.C.); johnpumc@gmail.com (Y.W.); duliqing@irm-cams.ac.cn (L.Q.D.); xuchang@irm-cams.ac.cn (C.X.); wangqin@irm-cams.ac.cn (Q.W.); fanfeiyue@irm-cams.ac.cn (F.Y.F.); fansaijun@irm-cams.ac.cn (S.J.F); 2 Tianjin Key Laboratory of Molecular Nuclear Medicine, Tianjin 300192, China; 3 First Clinical Department of Medical Emergency Response Centre for Nuclear Accident, Ministry of Health, Tianjin 300192, China; 4 Tianjin Third Central Hospital, Tianjin 300170, China; E-Mail: yadibaby1019@163.com; 5 National Institute for Radiological Protection and Nuclear Safety, Chinese Centre for Disease Control, Beijing 100088, China; E-Mails: jxlamy@gmail.com (J.X.L.); suxu@nirp.cn (X.S.); 6 Medical Emergency Response Centre for Nuclear Accident, Ministry of Health, Beijing 100088, China

**Keywords:** radiation accident, chromosome aberration, micronucleus assay, comet assay, DNA-DSB

## Abstract

The present study investigates cytogenetic damage in lymphocytes, derived from three victims who were unfortunately exposed to cobalt-60 (^60^Co) radiation (the 1999 accident occurred in a village in China’s Henan province). Case A of the three victims was exposed to a higher dose of ^60^Co radiation than Cases B and C. The chromosomal aberrations, cytokinesis-block micronucleus (CBMN, the CBMN assay), and DNA double-strand breaks (DSBs, the comet assay) examined in this study are biomarkers for cytogenetic abnormalities. After the lymphocytes collected from the victims were cultured, the frequencies of dicentric chromosomes and rings (dic + r) and CBMN in the first mitotic division detected in the lymphocytes of Case A were found to be substantially higher than in Cases B and C. Similarly, the DNA-DSB level found in the peripheral blood collected from Case A was much higher than those of Cases B and C. These results suggest that an acutely enhanced induction of the ^60^Co-induced cytogenetic abnormality frequency in humans depends on the dose of ^60^Co radiation. This finding is supported by the data obtained using practical techniques to evaluate early lymphoid-tissue abnormalities induced after exposure to acute radiation.

## 1. Introduction

On April 26, 1999, a radiation accident occurred in a village in China’s Henan Province. Briefly, radiation therapy equipment, including a ^60^Co radiation source that was purchased illegally. Three victims were exposed to gamma irradiation from the ^60^Co source. The ^60^Co (half-life of 5.27 years) radiation source was a metallic solid capable of emitting gamma rays, which are widely used for radiation therapy. Accidental exposure to gamma rays may cause various injuries to the human body. Many radiation sources, including ^60^Co, represent significant hazards to human health, and exposure to such sources has resulted in numerous injuries and deaths worldwide [1,2]. Hematological tissue, particularly lymphocytes, is one of the tissues that is most sensitive to ionizing radiation [3].

It is widely known that cytogenetic changes, including chromosome aberrations and the presence of micronuclei, are sensitive biomarkers for evaluating the damage caused by acute radiation exposure [4–7]. Specifically, dicentric chromosomes and rings (dic + r) are considered to be the standard marker for radiation exposure [8,9]. Cytokinesis-block micronuclei (CBMN) are another sensitive biomarker that can be used to estimate acute radiation damage and are widely used as a supplement to chromosome aberration analysis [10,11].

Single-cell gel electrophoresis, which is popularly known as the comet assay, and can quantitatively detect DNA breaks and repair kinetics at the level of a single cell, has been widely used in recent years in radiation biology, toxicology, oncology, and molecular epidemiology [12–16]. The comet assay may be a rapid and sensitive technique suitable for *in vivo* human biomonitoring, especially in cases of incidental exposure to ionizing radiation [17,18].

In the present study, we detected cytogenetic changes in the lymphocytes of the three individuals using chromosome aberration analysis and micronuclei assays, and we quantitatively analyzed DNA damage using the comet assay.

## 2. Results and Discussion

### 2.1. Chromosome Aberration in Leukocytes Derived from Three Victims

**Figure 1:**
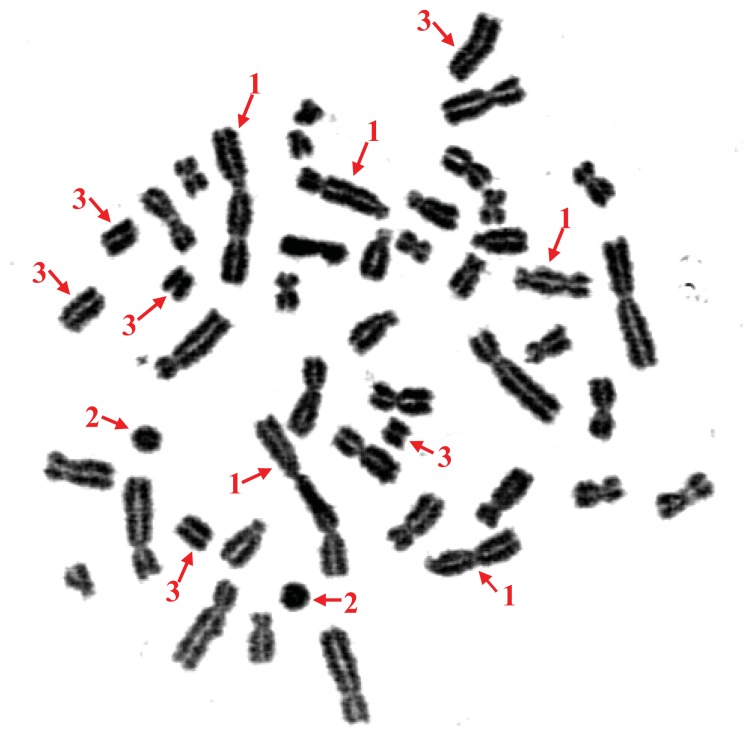
Multiple chromosome aberrations in a lymphocyte from Case A. **1**, dicentric chromosomes; **2**, centric rings; **3**, acentric fragments.

Tables 1 and 2, and Figure 1, show the cytogenetic effects of ^60^Co on humans.

The frequency of dic + r in Case A was significantly higher than that in Cases B and C (χ^2^ = 105.35, *p* = 0.000; χ^2^ = 81.04, *p* = 0.000, respectively), which demonstrates that Case A was exposed to a higher dose of radiation than Cases B and C. The aberration rate of Case C was slightly higher than that of Case B (χ^2^ = 3.88, *p* = 0.049). Table 1 also shows the biological dose estimation for each subject using the dose-response curve that was previously established in our laboratory for γ ray-induced chromosome aberrations (dic + r). The exposure dose for Case A was much higher than that of Cases B and C.

As shown in Table 2, the metaphase lymphocytes with multiple dic + r accounted for 60.7% of the total lymphocytes examined in Case A, whereas the proportions in Cases B and C were 12.3% and 11.0%, respectively. The findings indicate that more lymphocytes with multiple aberrations are observed in the subject who received higher doses of radiation. It should be noted that the number of lymphocytes with two or more aberrations contributed greatly to the assessment of radiation damage.

In the present study, chromosome aberration analysis was found to be useful in the assessment of cytogenetic toxicity *in vivo* after a high-dose, acute radiation accident. In the three victims of this radiation accident, we primarily observed dic + r aberrations, which are the distinctive aberrations used to assess radiation damage in lymphocytes. Gamma rays, which were emitted by ^60^Co in this accident, may cause chromosome breaks. The broken regions of separate chromosomes come into contact, and the ends move about and eventually combine to form exchanges. A dicentric chromosome occurs when there is an exchange between the centromeric pieces of two broken chromosomes, and in its complete form, it is accompanied by a fragment composed of the acentric pieces of these chromosomes (Figure 1). A centric ring chromosome occurs when there is an exchange between two breaks on separate arms of the same chromosome and is also accompanied by an acentric fragment. In human lymphocytes, centric rings are much more rare than dicentric chromosomes, and some researchers combine both forms for the assessment of radiation damage. Obvious aberrations, especially dic + r, were found in all three cases in the present study, and the aberration rate increased concurrently with the radiation dose. The results suggest that a significant increase in the frequency of the chromosomal aberrations is caused by the high dose of ^60^Co radiation, namely 5.61 Gy.

### 2.2. CBMN Assay in Three Victims

**Figure 2:**
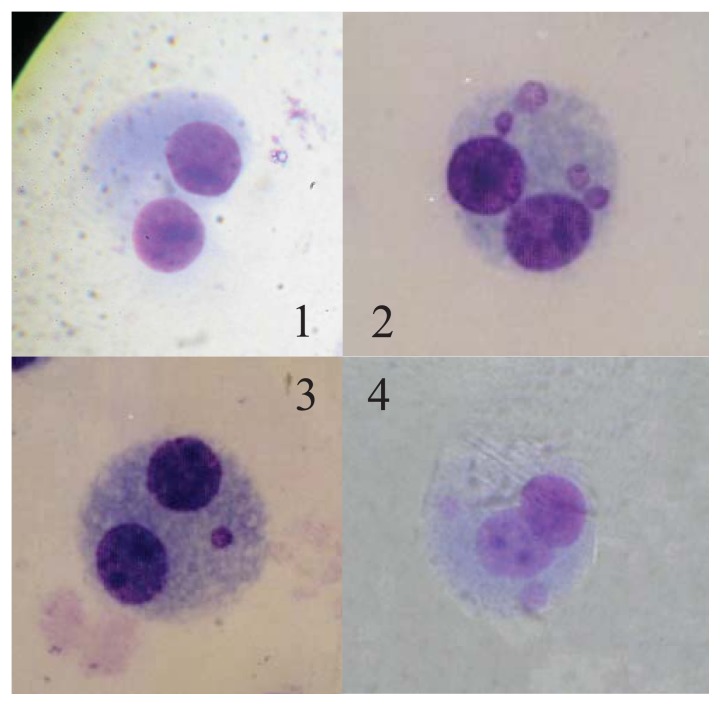
Cytokinesis-blocked micronuclei in lymphocytes from three cases of a radiation accident. **1**, A normal, binucleated lymphocyte; **2**, A binucleated lymphocyte with four micronuclei from Case A; **3**, A binucleated lymphocyte with one micronucleus from Case B; **4**, A binucleated lymphocyte with two micronuclei from Case C.

Table 3 and Figure 2 show ^60^Co-induced cyogenetic changes in the lymphocytes collected from the victims.

The frequency of CBMN was significantly higher in Case A than in Cases B and C (χ^2^ = 32.68, *p* = 0.000; χ^2^ = 30.72, *p* = 0.000, respectively), which also demonstrates that Case A was exposed to a higher dose of radiation than Cases B and C. No difference was found between Case B and C (χ^2^ = 0.092, *p* = 0.762). The results of the biological dose estimation by CBMN for all the cases are also included in Table 2. The dose estimation was also conducted according to the dose-response curve that was previously established in our laboratory for γ ray-induced CBMN. Fig 2 shows the CBMNs of Cases A, B, and C.

Gamma rays from ^60^Co can cause the formation of acentric chromosome fragments and the malsegregation of whole chromosomes. Acentric chromosome fragments and whole chromosomes that are unable to interact with the spindle lag behind at anaphase, and as a result, they are not included in the main daughter nuclei. A lagging chromosome fragment or whole chromosome forms in a small separate nucleus; hence, the term micronucleus [19]. Because of its good reliability and reproducibility, the CBMN assay has become a standard cytogenetic technique for genetic toxicology testing in human and mammalian cells in general. In the field of radiation protection, the CBMN assay for peripheral blood lymphocytes is an appropriate biological dosimetry tool for evaluating the *in vivo* radiation exposure of occupationally, medically and accidentally exposed individuals [20]. The scoring of micronuclei in BN lymphocytes provides an easy and rapid method for assessing cytogenetic damage and estimating the radiation dose. In the present study, the CBMN rate for each case was fully concordant with the dic + r rate determined by chromosome aberration analysis. Similarly, the biological dose estimation for the three cases, as assessed by CBMN, was consistent with that determined by dic + r, which suggests that both the CBMN assay and chromosome aberration analysis are capable of providing reliable cytogenetic damage assessment and accurate bio-dosimetry data within a certain period after acute external radiation [11].

### 2.3. DNA-DSBs Evaluated by Neutral Comet Assay

**Figure 3:**
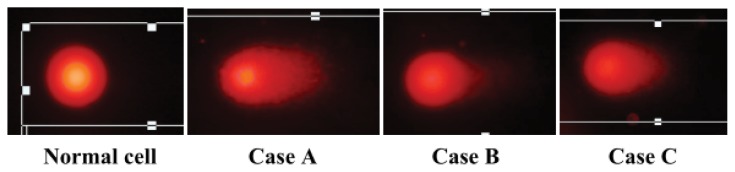
Comet images of lymphocytes from the three cases. The slides were stained with ethidium bromide, and the images were collected using a fluorescence microscope.

**Figure 4:**
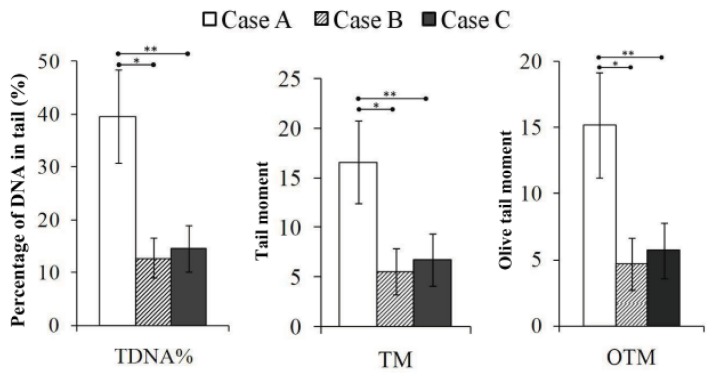
Quantitative analysis of residual DNA-DSBs in the lymphocytes of the three victims using the comet assay. *, Significant differences in TDNA%, TM and OTM were observed between Cases A and B, as analyzed by Student’s *t*-test, *p* = 0.000; **, Significant differences were observed between Cases A and C, as analyzed by Student’s *t*-test, *p* = 0.000; No difference was found between Cases B and C for all three comet parameters.

Figures 3 and 4 show ^60^Co-induced DNA damage of the lymphocytes. The comet assay is a sensitive method for evaluating DNA damage and has been used for the biomonitoring of individuals who have been accidentally exposed to ionizing radiation, either environmentally or occupationally [21]. Two versions of the comet assay exist. The alkaline version detects DNA single-strand breaks (SSBs), and the neutral version identifies double-strand breaks (DSBs) [22]. Among the various DNA lesions induced by gamma ray ionizing radiation, DSBs are considered the most important because of their potential to cause cell death, mutagenesis, and carcinogenesis [16]. Whereas DNA SSBs are usually completely repaired 24 h after irradiation, residual DSBs are usually still present at this time point [22]. DNA damage caused by ^60^Co exposure can be repaired, generating an apparently normal chromosome. Alternatively, such damage can be mis-repaired, resulting in an exchange, or remain unrepaired. The DNA DSB is the lesion responsible for most visible chromosomal aberrations observed at metaphase after lymphocyte irradiation [6]. Therefore, a neutral comet assay was performed to detect residual DNA-DSBs because the blood samples were obtained four days after the accident.

The comet images of lymphocyte nuclei derived from normal human and the three victims show the intensity of DNA damage (Figure 3). The comet tail length of Case A appeared longer than that of Cases B and C. As shown in Figure 4, the percentage of DNA in the comet tail (TDNA%), tail moment (TM) and Olive tail moment (OTM) of Case A were much higher than those of Cases B and C (*p* = 0.000), which demonstrates that the level of DNA damage in Case A was much higher than that in Cases B and C. No significant difference was found between Cases B and C.

The results of this study indicate that the comet assay is a sensitive method for detecting radiation-induced DNA damage, particularly for detecting DNA-DSBs in lymphocytes after ^60^Co gamma ray exposure. Studies of the comet assay have shown clear dose-response relationships in Chinese hamster ovary (CHO) cells [23], tumor cells [24,25], and germ cells [26] after irradiation. The comet assay is sensitive to the time at which the test is performed after radiation, and most of the DNA damage was repaired within 24 h after radiation. Although the level of DNA damage diminished during a one-year period, it remained higher than normal values recorded in the leukocytes of a healthy, unexposed person [17]. For each case in the present study, the residual DNA-DSB level determined by the neutral comet assay was fully consistent with the cytogenetic changes detected by the chromosome aberration analysis and CBMN assay. The extent of residual DNA damage in Case A was significantly higher than that in Cases B and C, and also showed an obvious dose-response relationship.

## 3. Experimental Section

### 3.1. Subjects

Three victims who were accidentally exposed to ^60^Co γ-rays were included in this study. Peripheral blood was sampled from the individuals on April 30, 1999, four days after the accident. Some information regarding the three victims is shown in Table 1. More details of the accident have been reported by Liu *et al.* [27]. Prior to the study, written informed consent was obtained from all subjects.

### 3.2. Reagents and Instruments

Cytochalasin B, colchicine and Giemsa stain were purchased from Sigma-Aldrich (St Louis, MO, USA). A Nikon 90i fluorescence microscope was purchased from Nikon (Tokyo, Japan). The chromosome image analysis system was purchased from United Scientific USA (Cherry Hill, NJ, USA) and the Sanyo MCO-20AIC CO_2_ incubator was purchased from SANYO (Sakata, Japan). The normal-melting-point agarose was obtained from Biowest (Kansas, MO, USA), and the low-melting-point agarose was from Promega (Madison, WI, USA). Tris-HCl, dimethyl sulfoxide (DMSO) and Triton X-100 were from Sigma (St. Louis, MO, USA). Lymphocyte-separated medium (lymphoprep) was purchased from Axis-Shield (Dundee, UK). The horizontal-strip electrophoresis apparatus was from Bio-Rad (Hercules, CA, USA). The ^137^Cs radiation source was from Atomic Energy (Chalk River, Ontario, Canada). The comet slides were from Biocomet. The digital imaging system was purchased from Union Science (Cherry Hill, NJ, USA).

### 3.3. Cell Culture and Sample Preparation

The blood samples were obtained four days after the accident, and all of the analyses were performed immediately after sampling. The procedures for lymphocyte culture were conducted according to the description in the IAEA-405 report [9]. Briefly, 0.3 mL of venous blood was collected from each subject four days after the accident using a heparinized syringe. The samples were added to 4 mL of RPMI-1640 medium containing 20% of bovine calf serum and 50 μg/mL PHA. The mixture was then incubated at 37 °C in a 5% CO_2_ incubator for 24 h. The blood cultures were then divided into two aliquots for the chromosome aberration analysis and micronucleus assay, respectively. For the chromosome aberration analysis, a final concentration of 0.06 mg/mL colchicine was added to the culture, and after culture for an additional 32 h, the lymphocytes were harvested, fixed, and stained with Giemsa, as previously described [9]. Dicentric chromosomes (dic) and rings (r) were scored under a light microscope.

For the CBMN assay, the cells were cultured for 44 h, after which a final concentration of 6 μg/mL cytochalasin B was added to the culture. Upon application of cytochalasin-B, lymphocytes that have completed one nuclear division accumulate and can be recognized as binucleate (BN) cells. The lymphocytes were harvested, fixed and stained with Giemsa 28 h later, as previously described [9]. Using the Giemsa-stained slides, the micronuclei were enumerated in 1000 bi-nucleated cells per subject [9].

### 3.4. Comet Assay

Whole blood (0.15 mL) was carefully layered in a ratio of 1:2 onto the lymphocyte-separating medium in a centrifugation tube. A gray layer of lymphocytes appeared between the blood plasma and the lymphoprep after centrifugation for 2 min at 3500 rpm. The lymphocytes were then carefully transferred to a 1.5 mL Eppendorf tube containing 1.2 mL of phosphate-buffered saline (PBS, 0.1 M) and centrifuged for 5 min at 2000 rpm. The lymphocytes were washed twice and resuspended in PBS at a density of (5–6) × 10^4^/mL. The cell viability was determined to be 98% by Trypan blue testing, and the lymphocyte suspension was stored in a refrigerator at 4 °C.

The comet assay was performed under neutral conditions, as described by Banath *et al.* [28]. The comet slides were coated with 100 μL of normal-melting-point agarose, and once the first agarose layer was coagulated, a mixture of 75 μL of low-melting-point agarose and 25 μL of lymphocyte suspension was applied as the second layer. The comet slides were immersed in cold fresh lysis solution (2.5 M NaCl, 1% *N*-sodium lauroyl sarcosinate, 30 mM Na_2_EDTA, 10 mM Tris, 1% Triton X-100 and 10% DMSO) for 1.5 h at 4 °C. After lysis, the slides were placed in buffer for 20 min in a horizontal electrophoresis tank pre-filled with cold fresh Tris-borate-EDTA buffer (TBE) to loosen the tight double-helical structure of the DNA for electrophoresis. Electrophoresis was then performed at 200 mA for 20 min in TBE buffer at room temperature. Following this, the slides were rinsed twice with distilled water and stained with ethidium bromide (2 μg/mL). All of the procedures were conducted in the dark to prevent supernumerary DNA damage. The comets were viewed using a Nikon 90i fluorescence microscope, and images of 400 comets were collected for each case using a digital imaging system. Overlapping cells were not counted. All comet images were analyzed using CASP software [29], and the percentage of DNA in the comet tail (TDNA%), tail moment (TM), and Olive tail moment (OTM) were recorded to describe the DNA damage to lymphocytes. All of the experiments were repeated twice. The variation between experiments was analyzed.

### 3.5. Statistical Analysis

The chi-squared test was used to compare differences in chromosomal aberration and micro-nuclear rate in the three cases using Crosstabs Analysis. The Student’s *t*-test was used to compare the difference in DNA-DSB levels in the three cases, and differences were defined as significant when *p* < 0.05.

## 4. Conclusions

The results of the present study suggest that an acutely enhanced induction of the ^60^Co-induced cytogenetic abnormality frequency in humans depends on the dose of ^60^Co radiation. This finding is supported by the data obtained using practical techniques to evaluate early lymphoid-tissue abnormalities after acute radiation exposure. A potential dose-effect relationship between cytogenetic abnormalities is found particularly in the early stage of radiation accidents.

## Figures and Tables

**Table 1:** Chromosome aberration analysis and biological dose estimation.

Subject	Sex	Age (years)	Number of lymphocytes examined	“dic + r”		Dose estimation (95% CI, Gy)

				Total count	Aberration rate (/cell)	
A	Female	38	150	357	2.38 *,#	5.61 (2.29~5.90)
B	Male	8	300	154	0.51 **	2.48 (2.26~2.68)
C	Male	37	300	178	0.59	2.68 (2.46~2.89)

Notes: dic + r, dicentric chromosomes and rings;

* compared with Case B, χ^2^ = 105.35, *p* = 0.000;

# compared with Case C, χ^2^ = 81.04, *p* = 0.000;

** compared with Case C, χ^2^ = 3.88, *p* = 0.049;

CI, confidence intervals.

**Table 2:** The distribution of “dic + r” in lymphocytes.

Subject	Number of dic + r per cell										

	0	1	2	3	4	5	6	7	8	9	Total
A	33	26	29	22	16	11	7	3	2	1	357
B	195	68	27	8	2	0	0	0	0	0	154
C	170	97	21	9	3	0	0	0	0	0	178

**Table 3:** CBMN assay and biological dose estimation.

Subject	Number of lymphocytes	Total micronuclei	Frequency of micronuclei (/cell)	Dose estimation (95% CI, Gy)
A	140	70	0.50*,#	5.45(4.28~6.62)
B	1000	265	0.265**	2.78(2.42~3.13)
C	1000	271	0.271	2.84(2.49~3.21)

Notes:

* compared with Case B, χ^2^ = 32.68, *p* = 0.000;

# compared with Case C, χ^2^ = 30.72, *p* = 0.000;

** compared with Case C, χ^2^ = 0.092, *p* = 0.762;

CI: confidence intervals.
